# Supplementary material for: Single-Molecule Insights into ATP-Dependent Conformational Dynamics of Nucleoprotein Filaments of Deinococcus radiodurans RecA
Source: Int J Mol Sci. 2020 Oct 7;21(19):7389. doi: 10.3390/ijms21197389 (PMC7583915; doi:10.3390/ijms21197389)
Supplement: Supplementary file 1 [file ijms-21-07389-s001.pdf]

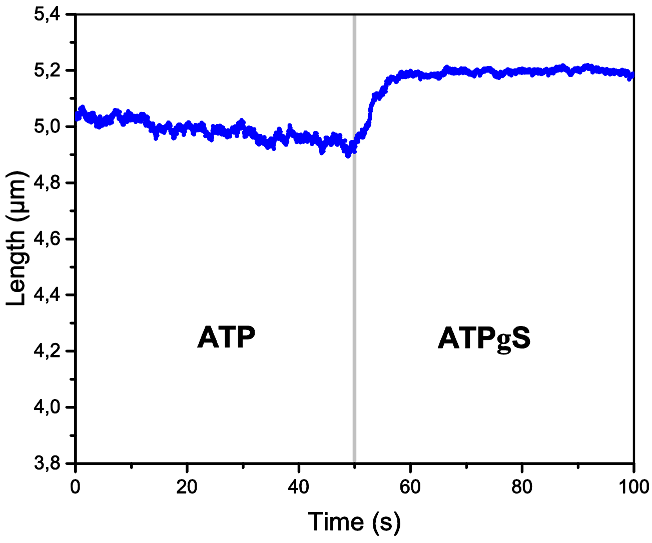

Figure S1. The change in the length of the preassembled DrRecA-ssDNA filament upon transition from the solution with 1 mM ATP to the solution containing 1 mM ATPγS. During measurements a constant tension of 3 pN was applied to the tether. The change in length was observed in the absence of free DrRecA in solutions.

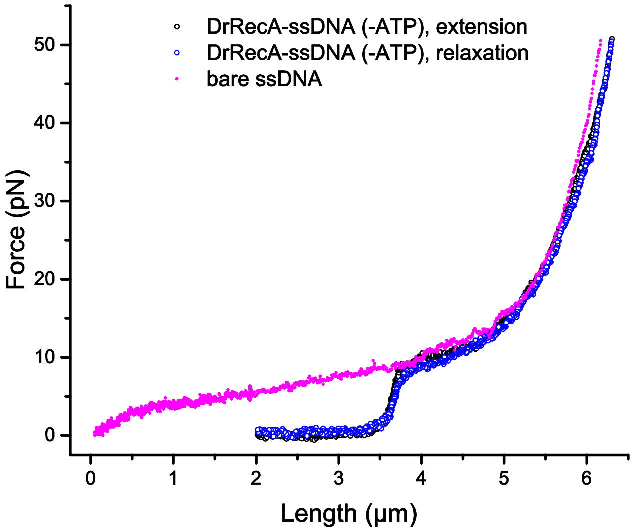

Figure S2. Force-extension behaviour of the compressed DrRecA-ssDNA filament (black – stretching, blue – relaxation). Force-extension behaviour of bare ssDNA is shown in magenta.

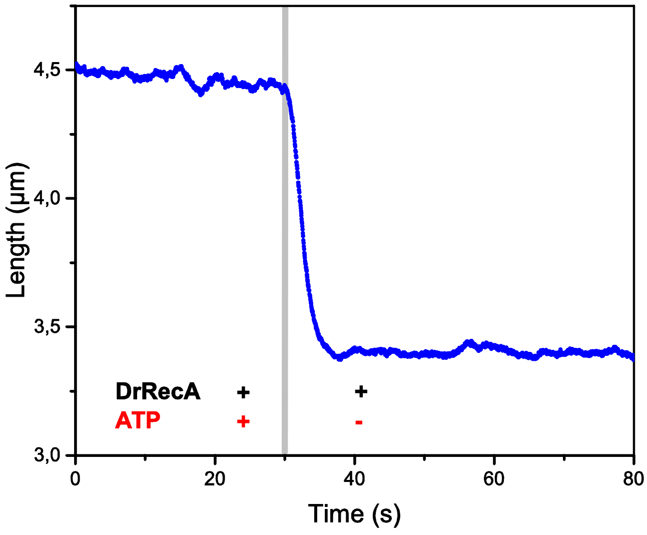

Figure S3. Dynamics of the DrRecA-dsDNA filament length upon transition from the channel containing both DrRecA and ATP to the channel containing DrRecA and no ATP. During transition a constant tension of 3 pN was applied to the filament. Experiment was carried out at 22°C.

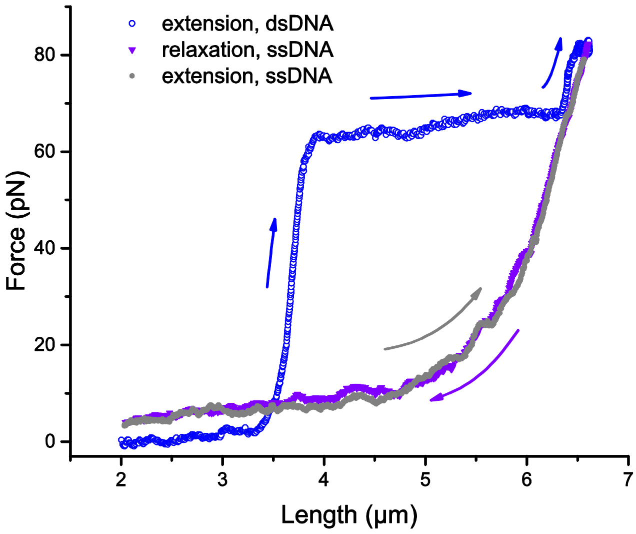

Figure S4. Generation of ssDNA by force-induced melting. To generate ssDNA, duplex DNA was stretched with a force above 80 pN for ten seconds (blue). Relaxation curve indicates that dsDNA was converted to ssDNA (violet). Subsequent stretching of the tether verifies that dsDNA was fully melted into ssDNA (grey).
